# Sensor Design Optimization for Ultrasonic Spectroscopy Cure Monitoring

**DOI:** 10.3390/s18061819

**Published:** 2018-06-04

**Authors:** Christian Pommer, Michael Sinapius

**Affiliations:** Institut for Adaptronics and Function Integration, Technische Universität Braunschweig, Langer Kamp 6, 38106 Braunschweig, Germany; m.sinapius@tu-bs.de

**Keywords:** ultrasonic, cure monitoring, resonant ultrasonic spectroscopy

## Abstract

In the field of cure monitoring, resonant ultrasonic cure monitoring is a unique technique to measure the progression of cure of composites in fully or partially closed tools. It allows for the use of electronic hardware that is less sophisticated than traditional pulse-based ultrasonic systems to obtain accurate results. While this technique is not new, it has been used very rarely. One reason for this is the lack of optimized sensors. Commercially available sensors are optimized for pulse-based ultrasonic testing. This paper establishes a possible optimized sensor design for resonant ultrasound cure monitoring using a multi-parameter FE model.

## 1. Introduction

Resonant ultrasonic spectroscopy (RUS) is a technique whereby the response spectrum of a harmonic or chirp excited specimen to gain information on its material properties is analyzed [1]. This technique is normally used to gain information on the specimen itself but can also be used to gain information on the change in boundary conditions, e.g., the change in acoustic reflection during the curing of an epoxy applied to the specimen. This method allows wear-free cure monitoring by monitoring the change in resonances within a tool, specially those resonances created between actuator and epoxy. If edge reflections are disregarded, the excited eigenmodes should be concentrical in nature. The amplitude of those resonances are directly related to the current state of cure and can be measured via an additional sensor. This method is described in greater detail in [2].

The principle of RUS-based cure monitoring is similar to pulse-based ultrasound-based density measurement described in the work of Puttmer et al. [3] and in the work of Kazys et al. [4] for pulsed ultrasonic. The differences lie in its execution and excitation method.

Cure monitoring has gained significant interest in recent years due to the growing significance of fiber composite materials in a wide range of industrial sectors. While composites are already well established in the aerospace industry, decision-makers in industrial sectors such as automotives are increasingly interested in using composite materials due to their low weight and high stiffness.

One of the fastest methods of producing composite structures is the pultrusion process. This method produces large quantities of slender structures at a high rate, with up to 10 m/min extrusion rates, at nearly the same costs as aluminium extrusion and with low material losses.

Although pultrusion is already highly automated, in-line cure monitoring systems are not yet state of the art in this process due to the difficult environment. There are, for example, currently no commercially available in-line or in-tool cure monitoring systems for the pultrusion process.

RUS can be used to gain information on the current state of cure of epoxy systems inside fully or partially closed tools, where the latter are common to the pultrusion process. A big advantage of RUS compared to other non-ultrasonic cure monitoring techniques in pultrusion is the non-direct contact and therefore wear-free operation. A wear-free sensor design is especially important for the pultrusion process, where long production sessions and an extreme rough environment in the tool, with high abrasion rates and temperatures up to 250 ${}^{\circ }$C, are common.

**Figure 1:**
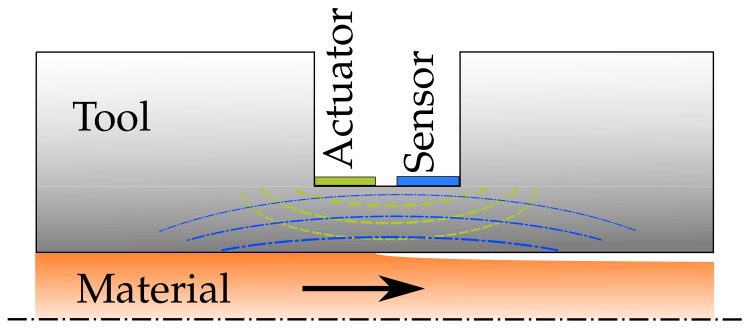
Actuator and sensor design inside a pultrusion tool before optimization.

Although the fundamentals of this technique for cure monitoring in industrial processes was established in [2,5], there are still several challenges that have to be addressed. An especially troublesome challenge is the lack of sophisticated sensors and sensor design guidelines for RUS cure monitoring. There are a number of recent articles that deal with ultrasound sensor design, such as [6,7], but they do not take the response function of the tool into account. While the principle itself allows for the use of crude sensors, for example two piezo ceramics applied to the tool surface as shown in Figure 1, to measure the curing of epoxies, as shown in [2], they are prone to acoustic reflections and do not fully incorporate a possible concentric eigenmode induced by the RUS.

Commercially available and classic ultrasound sensor heads are no alternative as they are optimized for pulse-based ultrasound techniques. They are normally not directly coupled to the tool, which creates additional impedance differences and diminishes the results, or they create extra noise as well as additional resonances.

There are in total three papers that use resonant ultrasonic spectroscopy to detect the curing of adhesives [2,5,8]. None of those articles analyzes the near-field behavior to optimize a specific sensor design. While there are already sophisticated measurement systems for ultrasonic spectroscopy [9,10,11], they are highly sophisticated and not designed to be used in unknown tool-sensor configurations.

**Figure 2:**
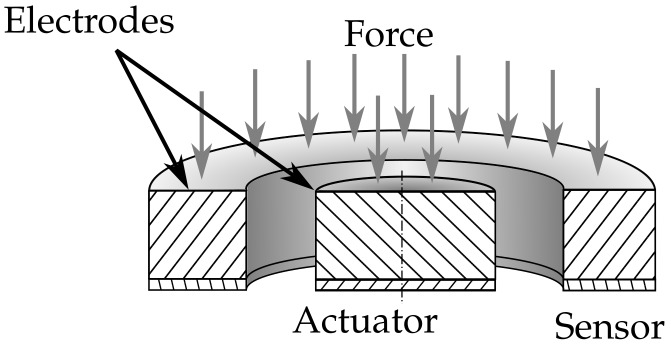
Alternative sensor design for more robust resonant ultrasonic cure monitoring measurements.

The study presented in this paper sheds additional light on the nature of RUS for cure monitoring. The near-field eigenmode of a steel plate was analyzed via laser Doppler velocimetry as well as FE modeling. The tuned FE model was then used to optimize a concentric sensor design for a given tool thickness to utilize a concentric eigenmode around a central actuator. Figure 2 illustrates this design.

This design enhances the sensitivity to concentric waves and reduces the sensitivity to non-concentric waves. The experiment yields a design rule for an optimal sensor layout in combination with an FEM simulation.

While similar techniques to measure the eigenmodes of plates have been used for structural health monitoring with guided ultrasonic waves [12,13], they are usually only used for thin plates and not for analyzing close-range eigenmodes. Even articles about thick plates [14] do not consider the possibility of a sensor in the acoustic near field of the actuator. This article specifically targets thick plates with an actuation used for RUS cure monitoring.

## 2. Materials and Methods

**Figure 3:**
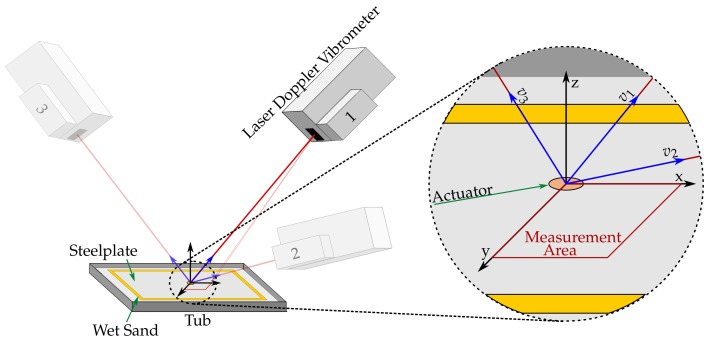
Experimental setup for different laser Doppler velocimeter (LDV) positions (1–3), with global Cartesian in-plane coordinate system N (x,y,z), local oblique measurement coordinate system K ($v_{1},v_{2},v_{3}$), and measurement area (red).

As stated above, the experimental setup consists of a scanning laser Doppler velocimeter (LDV) measuring the surface velocities of a 500 × 500 × 10 mm steel plate. The setup is displayed in Figure 3.

The measured surface velocities of the steel plate were used to tune a parametric FE model. A design rule was derived for different plate thickness’s and actuator diameters. The steel plate represents the pultrusion tool and the 10 mm thickness of the steel plate represents the targeted distance between the sensor and material in a typical pultrusion tool. While the size of the plate already reduces reflections as a result of the inherent material damping, the edges of the plate are additionally damped with a mixture of water and sand to suppress the boundary reflection. A piezoelectric actuator is placed in the center of the steel plate using an epoxy adhesive. The steel plate is decoupled by a soft foam underneath.

The 1D-LDV measures surface velocities in the direction of the laser-beam. Quite often, a single direction is sufficient to obtain all required information. In the case of RUS cure monitoring, 3D information may be required to accurately simulate an arbitrary sensor response with different means of surface attachment. A three-dimensional measurement allows for an assessment of the influence of the in-plane movement in the sensor signal.

A three-dimensional LDV measurement requires three linear independent measurement directions. This can usually be done by three independent LDV sensor heads. An alternative is to use a single sensor-head, and three separate measurements in three linear independent directions. The three-dimensional movement of one point is then obtained by combining the complex response spectra of the three separate measurements in its three independent directions (*v*1–*v*3). Those three linear independent measurement directions create an oblique coordinate system for every measurement point (blue). To ensure that all spectra can be combined independent from their time of capture, a harmonic stimulation with a source triggered oscilloscope is used.

A reflective spray improves laser reflectance. This, however, creates unexpected noise, as an alternative reflective paper creates a more even surface. However, the adhesive possibly decreases in-plane movement.

The first points of the measurement are used as geometrical references to ensure that all measurements can be geometrically aligned. Their order and physical locations are the same for all measurement directions. The reference point positions and the laser origin of the three measurements with their individual directions are transferred to a Cartesian coordinate system (x,y, and *z*), which aligns with the plate dimensions (black).

The measured excitations are then used to to determine the influence of in-plane movement and to tune the FE model of this experiment. This FE model is the basis for simple geometric sensor optimization for a concentric sensor design.

## 3. Results

**Figure 4:**
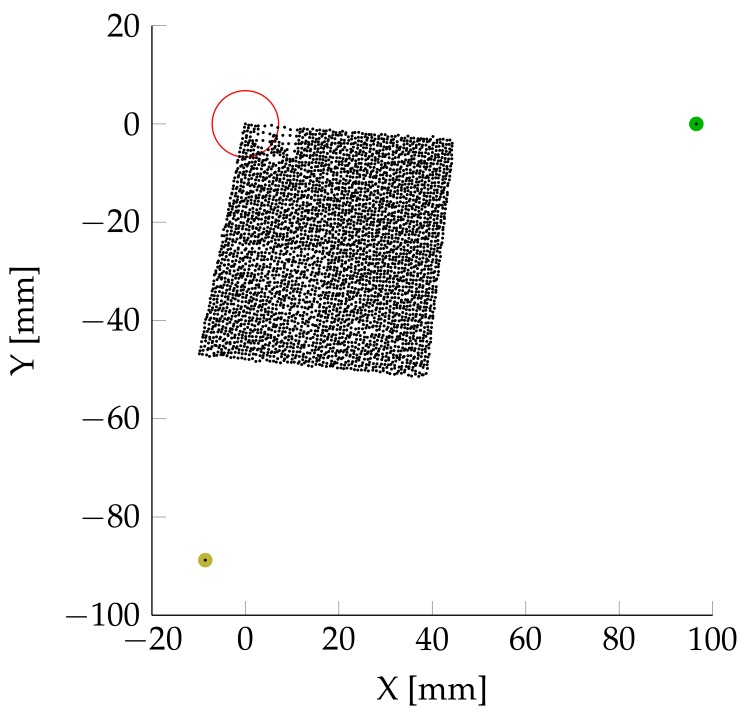
Point distribution. Actuator: red; geometrical reference points: green and yellow.

Figure 4 shows the measurement point distribution for one LDV measurement direction in the Cartesian in-plane coordinate system. The actuator (red) is placed in the center of the coordinate system. The first measurement point defines the origin. The second measurement point (green) defines the direction of the *x*-axis. The third measurement point (yellow) defines the *x*–*y* plane and the direction of the *y*-axis. The visible rectangular area with the vast majority of measurement points has a dimension of 50 × 50 mm. This rectangle represents one quadrant of the propagating waves. One quadrant is sufficient, as there is no extensive angular dependency. A non-circular symmetric result would be an indicator for unwanted boundary reflections at the plate’s outer edges. The tilt of the points positions is a result of the reference point distribution. It has, however, no influence on the results itself and is therefore disregarded.

**Figure 5:**
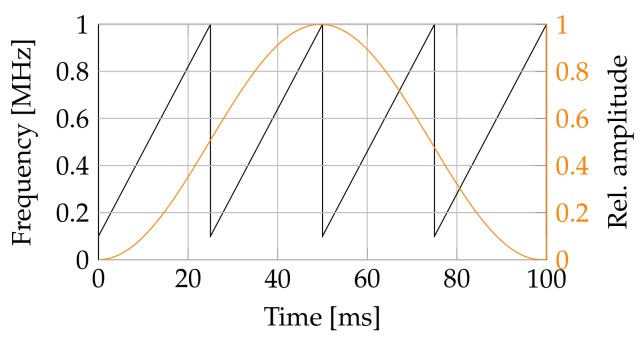
Frequency curve as well as window function during a single measurement.

In order to obtain an accurate amplitude spectrum, a quasi-harmonic excitation with a swept sine signal ranging from 100 kHz to 1 MHz is used for stimulation. The swept sine has a time length of 25 ms. Four of those swept sine signals were fit together for a single measurement. A cross spectrum was created between output and input. To minimize the boundary effects, a Hanning window function was applied to weigh the output and input signal. Figure 5 shows the frequency over time as well as the window function for a single measurement. The cross power spectra of 10 windowed swept sine blocks were averaged by their spectral power to create the final cross power spectrum for each measurement point. In order to minimize noise, only frequencies of the response with a coherence factor above 0.9 were used. No additional filter, except the built-in anti-aliasing filter of the measurement instrument, was used.

**Figure 6:**
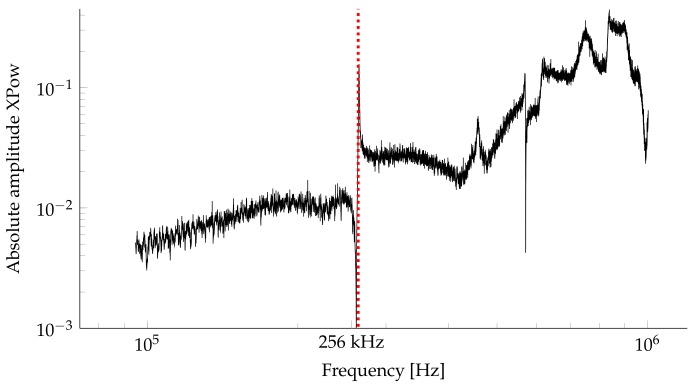
Mean absolute amplitude spectrum of 10 individual cross spectra with first resonance frequency in the thickness direction (red).

Figure 6 shows the amplitude distribution of the resulting averaged cross power spectrum.

The sharp resonance frequency at 264 kHz indicates the first mode shape of the plate in its thickness direction. This can be confirmed by calculating the acoustic wavelength, which is twice the plate thickness at this frequency.

**Figure 7:**
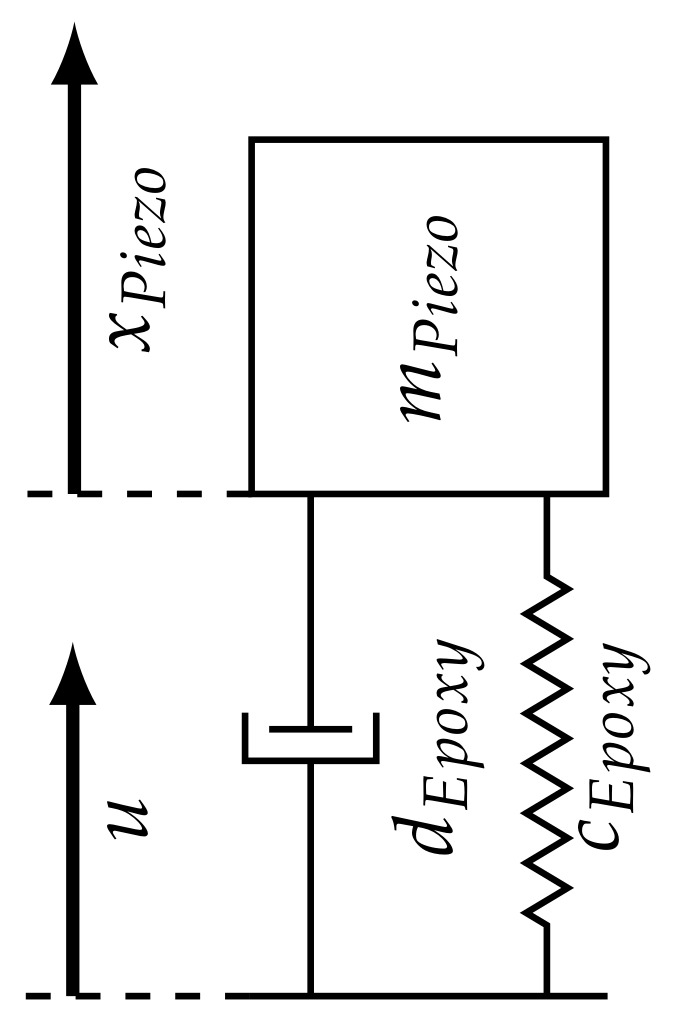
Spring–mass–damper model of the piezo-actuator and epoxy.

Furthermore, a steady increase in amplitude, starting at 500 kHz and peaking at around 900 kHz, is visible. A possible reason for this is an increase in the power of the actuation. The actuator and the epoxy create a mass–spring–damper system, schematically shown in Figure 7, with a distinct resonance frequency. The power of the actuation system is amplified proportionally with the closeness of the excitation and resonance frequency. Anregung

The spring–mass–damper system has the parameters of Table 1.

**Figure 8:**
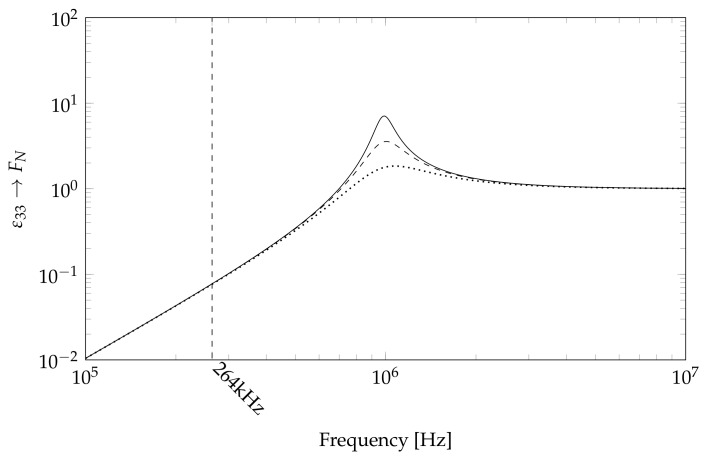
Simulated cross spectrum for the correlation of piezo-strain and normal force on the plate with different Lehr’s damping measures: solid 0.050; dashed 0.10; dotted 0.20. Scaled to $F_{N}\left(\omega_{\infty }\right)$.

The damping is unknown, so a range of different damping values is assumed based on a rough estimation of the real damping value for RTM6. The values of Table 1 yield the cross power spectrum displayed in Figure 8.

Figure 8 confirms the assumption stated above of a resonance at around 900 kHz. It is clearly visible that the damping value has a high influence in this region. However, for the resonance frequency in question at 264 kHz, there is nearly no influence of the damping value.

The resonance is visible in Figure 6 as a general increase in amplitude. Figure 6 does not show a single resonance amplitude. Instead, it shows a general amplification of resonance amplitudes, which is caused by the spring–mass–damper behavior of the actuator mounting. The resonance of the actuator–epoxy system creates a higher excitation force, amplifying other system resonance frequencies. This behavior could be used to gain higher amplitudes in the resonance regions by manipulating the epoxy thickness or by adding mass to the actuator during application.

**Figure 9:**
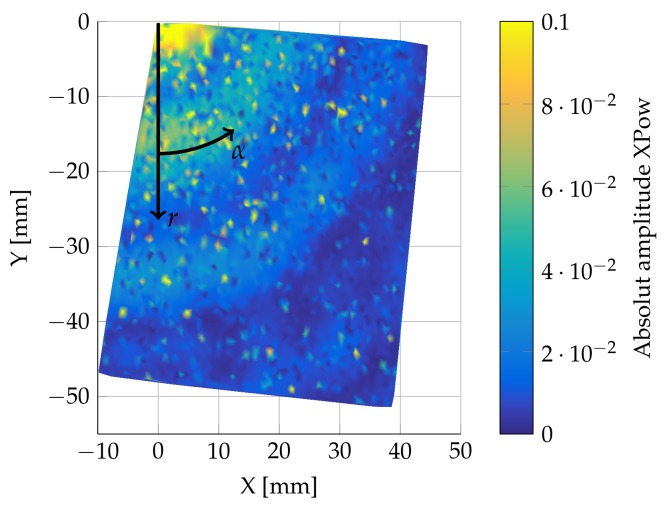
XPOW absolute amplitude distribution at 264 kHz.

This article focuses on the first resonance frequency at 264 kHz. Figure 9 shows the absolute amplitude of the cross power spectra at 264 kHz for all given measurement points of one direction on the surface of the steel plate.

A concentric distribution of the amplitudes is clearly visible, which indicates the desired infinite plate response. The visible ripples are an unexpected result of an overlap of different wave propagations. This structure with slightly different ring diameters is visible in all three directions.

**Figure 10:**
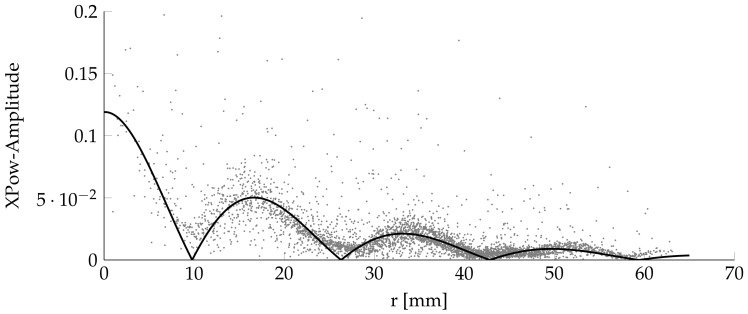
Amplitude approximation of the equation to measured values.

Figure 10 displays the absolute amplitude *A* over distance from the center *r*. The ripples in Figure 9 are more visible in this format. These ripples are a mixture of standing and traveling waves.

For better readability and processibility, the data points are fitted with Equations (1). The equations are based on observation. The response of the amplitude *A* can be described by

(1a) $$A=|A_{0}\cdot \cos\left(r\cdot 190+n\right)\cdot e^{-r\cdot c}\cdot \left(1+\alpha \cdot m\right)|$$

(1b) $$r=\sqrt{{\left(x+dx\right)}^{2}+{\left(y+dy\right)}^{2}}$$

(1c) $$\alpha =\arctan2\left((x+dx),(y+dy)\right).$$

x,y are the Cartesian point coordinates, with correction factors dx, dy. α is the angle between the *x* axis and the point position, while *r* is the radius. α and *r* are the coordinates of the cylinder coordinate system. $A_{0}$, *n*, *c*, and *m* are fitting factors.

The least square fitting is done in all three directions. An adjusted coefficient of determination approach is used to determine the optimal fitting factors. Table 2 contains the received fitting factors, as well as the adjusted coefficient of determination $R^{2}$.

The phase φ shows a circular pattern as well. The phase fit is done by a numerical approach based on the following equations:

(2a) φ=(r·b+c+d·sin(r·e+f))

(2b) $$r=\sqrt{{\left(x+dx\right)}^{2}+{\left(y+dy\right)}^{2}}$$

(2c) $$\alpha =\arctan2\left((x+dx),(y+dy)\right).$$

**Figure 11:**
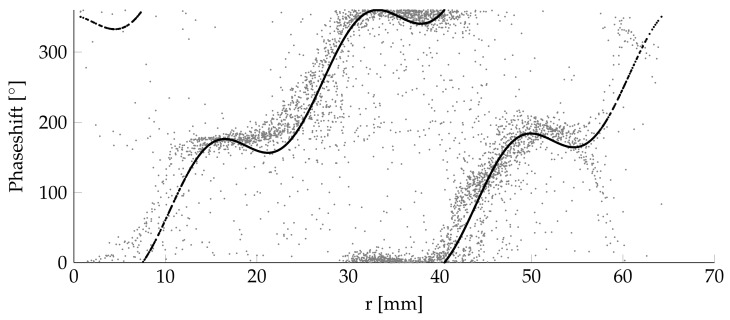
Phase approximation of the equation to measured values.

The result is shown in Figure 11.

Equation (2a) is similar to Equation (1a) but uses the fitting factors *b*, *c*, *d*, *e*, and *f*. An adjusted coefficient of the determination approach is used here as well to determine the optimal fitting factors. The fitting factors of those equations are shown in Table 3.

Using the fit results of Table 2 and Table 3, the movement of each point in all three measurement directions is calculated. As described above, an oblique coordinate system *K* is created for each point using the three measurement vectors of the LDV as base vectors $v_{1}$ to $v_{3}$. The complex amplitudes for each direction *a*, *b*, and *c* represent the excitation. This creates a three-dimensional excitation ${\vec{A}}_{K}$

(3) $${\vec{A}}_{K}=a{\vec{v}}_{1}+b{\vec{v}}_{2}+c{\vec{v}}_{3}.$$

The first step to transfer the excitation of this local oblique coordinate system to its representation in a global Cartesian coordinate system *N*, with the base vectors *x*, *y*, and *z*, is to represent the base vectors of the oblique coordinate system as vectors in the new Cartesian coordinate system.

(4) $$\vec{v_{i}}={\left(\begin{array}{c} x_{i}\\ y_{i}\\ z_{i} \end{array}\right)}_{N}$$

The transfer matrix [M] transfers the excitation of each point in the Cartesian coordinate system ${\vec{A}}_{N}$ to its oblique counterpart ${\vec{A}}_{K}$:

(5a) $${\vec{A}}_{K}=\left[M\right]{\vec{A}}_{N}$$

(5b) $$\left[M\right]={\left[\begin{array}{ccc} x_{1} & x_{2} & x_{3}\\ y_{1} & y_{2} & y_{3}\\ z_{1} & z_{2} & z_{3} \end{array}\right]}_{N}$$

By inverting the transfer matrix [M], the Cartesian representation of every three-dimensional excitation in the oblique coordinate system is calculated.

(6) $${\vec{A}}_{N}={\left[M\right]}^{-1}{\vec{A}}_{K}$$

**Figure 12:**
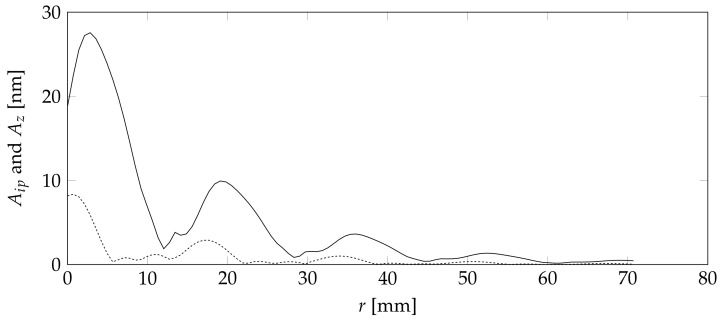
Calculated in-plane amplitude (dashed) and out-of-plane amplitude (solid).

Figure 12 shows the absolute in-plane $A_{ip}$

(7) $$A_{ip}=\sqrt{A_{x}^{2}+A_{y}^{2}}$$

as well as the absolute out-of-plane amplitude $A_{z}$ values for the newly created Cartesian coordinate system in dependence of the distance from the center *r*.

Figure 12 clearly shows the dominance of the out-of-plane movement with an excitation three times higher than that of the in-plane direction. This is a good indication that a sensor design based on a friction-based tool sensor contact, which might lose some of the in-plane movement, is not vastly inferior to a glued solution. The glued solution is less desirable due to the more difficultrepair.

Aside from the difference in amplitude, both directions clearly show the number of local minima at around 1.2, 2.9, and 4.5 cm. Those minima are are directionally independent. This is a clear indication that a geometric optimization will create discrete optimal points.

The use of an FE model has the advantage that it can incorporate the influence of the sensor similar to Figure 2.

**Figure 13:**
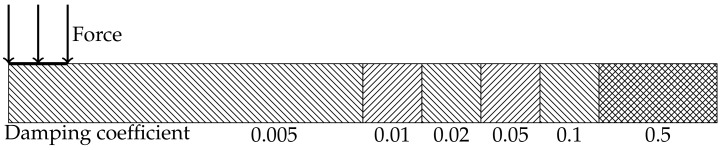
2D FEM model for the simulation of wave propagation in an endless plate.

As a result of the observed concentric nature of the response, a 2D FE model is a good trade-off between simulation accuracy and speed. The response of the created model was then compared with the experimental result to determine the accuracy of the model and adjust it if necessary. The simulation of a homogeneous plate with a response similar to an endless plate at moderate to low material damping values would create large simulations with long simulation times, where large areas of the simulated material would be unused. The application of a simulation consisting of the same material, with damping parameters increasing with the distance outside of the measurement area, can compensate for such problems. In this model, a center region has the same model parameters as the real plate. This region represents the measurement area and is slightly larger than the real measurement area. In this case, the center region has a radius of 100 mm. Additional rings are added to the simulation to remove any reflections at the region boundaries. Those rings have the same basic material properties but have increasing material damping. This method allows for the reflection-free wave propagation with much smaller models. Figure 13 illustrates this principle. The numbers below the segments are the damping coefficients. Each segment except the 0.5 segment and the 0.005 segment has a width of 10 mm. The 0.5 segment has a width of 30 mm to effectively remove all incoming waves. The force is applied as a harmonic surface load with a radius of 8 mm.

**Figure 14:**
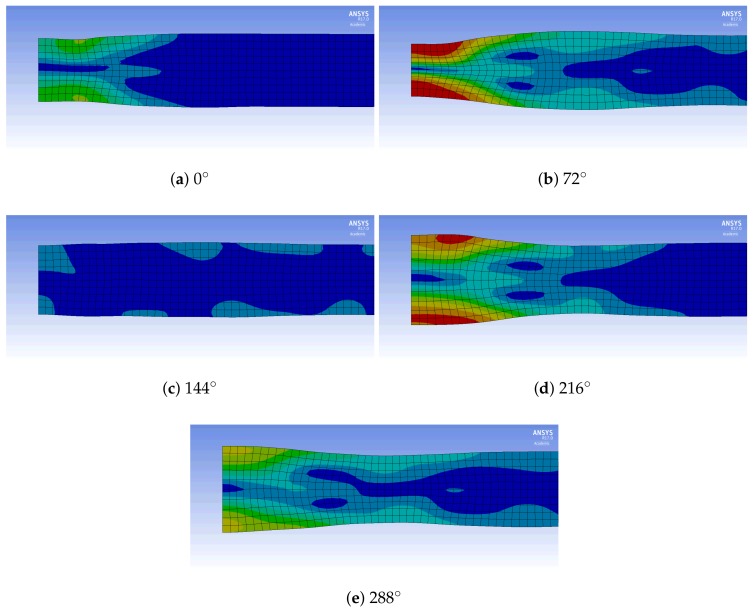
Total deformation at different phase angles (red maximal deformation and blue minimal deformation) at 271 kHz; element edge length: 1 mm; left edge: rotational axis.

**Figure 15:**
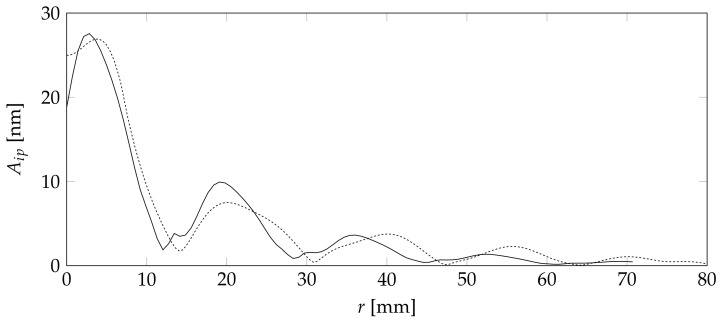
Simulated out-of-plane amplitude (dashed) and measured out-of-plane amplitude (solid).

The model uses a harmonic analysis and a rectangular mesh with a 1 mm edge length. Figure 14 displays the results of the simulation for the first resonance frequency in the thickness direction. The simulated resonance has a frequency of 271 kHz. The reason of the slight variation are most likely differences in material parameters. Figure 14 clearly shows a concentric ring structure similar to Figure 10, where some points have very little vertical movement. Figure 15 compares the simulated and measured vertical displacement of the steel plate and the FE model.

The figure clearly shows that the results of the measurement and the simulation are very similar. The ripples are visible in both curves. They are a result of overlapping traveling waves with different modes and speeds. The small difference between simulation and measurement might be a result of the nature of the steel plate. While the steel plate is simulated as an isotrope, in reality this is not entirely true due to the manufacturing processes used to create it. It is highly likely that the steel plate has an anisotropic behavior due to rolling. Nonetheless, the results are close enough that the anisotropy can be disregarded.

**Figure 16:**
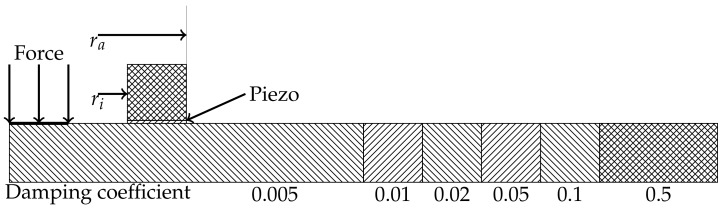
2D FEM Model for simulation of wave propagation in an endless plate.

To simulate an accurate sensor response for the optimization purposes, a sensor model consisting of a sensoric element and a backplate is added to the FE simulation Figure 13. The deformation of the sensoric element is then used to calculate the sensor response for a given geometry. The expanded simulation model is shown in Figure 16.

The simulated sensoric element has a thickness of 0.2 mm, and the adhesive connection between the sensor and the backplate is disregarded in the simulation. The back-plate is holding down the sensoric element. It is modeled as a metal ring structure with a high inherent damping of 0.5. It has the same acoustic impedance as the plate to absorb incoming acoustic waves. By varying the inner and outer radius of the sensor, an optimal sensor design for the given actuation and plate thickness with regard to a maximum sensor signal is achieved.

Equations (8a) are used to simulate the response $Q_{Total}$ of the sensoric element. The charge is used because the voltage might give a false impression. A small piezoelectric sensor has little capacity to create extraordinary high voltages, even at small charges that are heavily influenced by parasite capacities in the measurement equipment. Larger piezo sensors may create smaller voltages but are less influenced by the measurement equipment.

(8a) $$\begin{array}{cc} Q_{Total} & =Q_{\varepsilon_{x,y}}+Q_{\varepsilon_{z}} \end{array}$$

(8b) $$\begin{array}{cc} Q_{\varepsilon_{z}} & =Y_{z}\cdot d_{33}\cdot \underset{A}{\;\int \int \;}\left[\varepsilon_{z}\left(x,y\right)\right]dxdy \end{array}$$

(8c) $$\begin{array}{cc} Q_{\varepsilon_{x,y}} & =Y_{xy}\cdot d_{31}\cdot \underset{A}{\;\int \int \;}\left[\frac{\Delta x(x,y)}{dx}+\frac{\Delta y(x,y)}{dy}\right]dxdy. \end{array}$$

Equation (8a) forms the basis for an optimization of the geometrical parameters to maximize the created charge. They use Young’s modulus of the piezoceramic in the in-plane direction $Y_{xy}$ and in the out-of-plane direction $Y_{z}$ as well as the piezoelectric charge coefficient for the in-plane direction with the out-of-plane charge generation $d_{31}$ and the out-of-plane direction with the out-of-plane charge generation $d_{33}$. Furthermore, the equation utilizes the out-of-plane strain and the in-plane displacement Δx and Δy.

**Figure 17:**
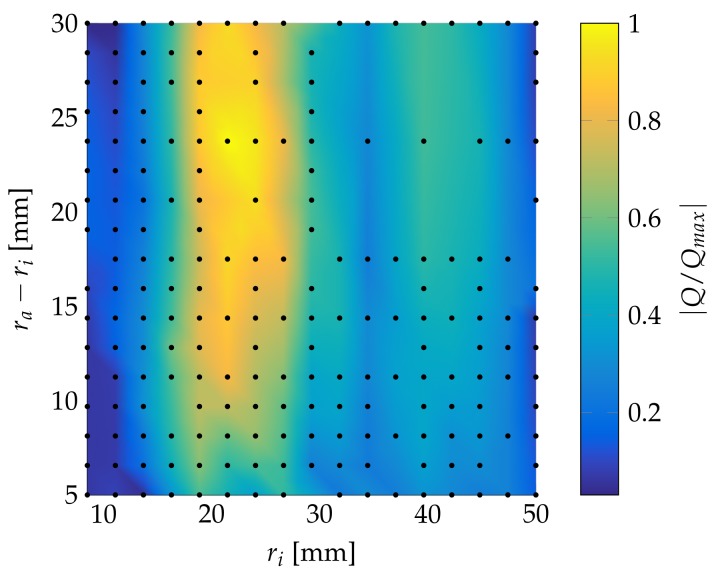
Relative generated electrical charge of a ring sensor with different geometries and a 0.2 mm thickness for a 5 mm diameter actuator and a 10 mm steel plate.

By simulating different sensor geometries, it is possible to obtain a better sensor design. Figure 17 displays the normalized generated electric charge for variable width and inner diameter.

Figure 17 clearly shows an optimal inner radius between 20 and 25 mm, with a sensor width also of 20–25 mm.

The sensor design optimization, however, has to be performed with the tooling used in pultrusion in mind as well as its process parameters. Large sensors may disrupt the heat flow inside those tools, resulting in deformed parts due to uneven curing. The optimal sensor parameters presented above are quite high for many tools and would have a high chance of disrupting the heat flow. A compromise between signal strength and sensor size needs to be found. A sensor with an inner diameter of 18.8 mm and a width of 5 mm still generates 60% of the maximum generated charge. This sensor, however, is still quite large.

**Figure 18:**
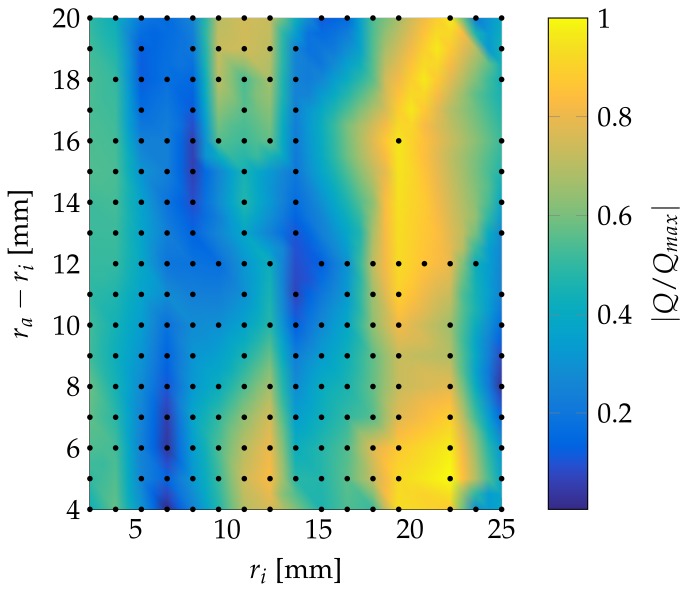
Relative generated electrical charge of a ring sensor with different geometries and 0.2 mm thickness for a 5 mm diameter actuator and a 5 mm steel plate.

Another approach is to reduce the distance between sensor and material to 5 mm. This generates the charge distribution shown in Figure 18. This allows the use of a far smaller sensor of only 12 mm inner radius and a 4 mm width. Due to the smaller size, the disruption in heat flow will decrease.

## 4. Discussion

Here we analyzed three parameters for the optimization of a sensor design similar to Figure 2, the inner sensor diameter, the outer diameter, and the actuator–epoxy distance. With the results found, it is now possible to create a concentric sensor design for a given actuator diameter. The actuator–epoxy distance should be kept to 5 mm. This distance allows for the use of smaller sensors. The optimal inner diameter for a sensor is simulated at 8 mm with a 24 mm outer diameter.

The ring sensor design reduces the overall undesired reflection sensitivity by being highly sensitive to concentric waves. Reflections at tool boundaries, except for the tool material boundary below the actuator, are not concentric, so they induce only minor charge in the sensor. To further reduce the sensitivity of non-concentric waves, the sensor can be divided in two half rings. It is possible to create a dedicated measurement system, especially for this type of sensor, by polarizing them in opposite directions and treating the signal of both sensors as a single differential signal. It is possible to create an output signal that further reduces all non-concentric wave parts. With this design, further investigations regarding the optimal sensor design have to be made. Furthermore, whether it is possible to reduce wave propagation outside the sensor area needs to be determined.

## Figures and Tables

**Table 1:** Material parameters [15,16].

Dimension	Value	Unit
$E_{Piezo}$	$4.83\cdot 10^{10}$	(N/m${}^{2}$)
$E_{Epoxy}$	$2.98\cdot 10^{9}$	(N/m${}^{2}$)
$\rho_{Piezo}$	$7.80\cdot 10^{4}$	(kg/m${}^{3}$)
$d_{Epoxy}$	200	(μm)
$d_{Piezo}$	50	(μm)

**Table 2:** Fit results amplitude measurement.

Units	$A_{0}$	c	m	n	dx	dy	$R_{\mathrm{adjust}}^{2}$
		(m${}^{-1}$)	rad${}^{-1}$		(mm)	(mm)	
Direction 1	0.124	52.2	−0.046	−0.29	−1.97	−0.50	0.93
Direction 2	0.180	66.8	−0.020	2.25	−3.46	−0.51	0.96
Direction 3	0.190	68.0	−0.027	2.18	−1.35	3.02	0.96

**Table 3:** Fit results phase measurement.

Units	b	c	d	e	f	$R_{\mathrm{adjust}}^{2}$
	$\left(\frac{{}^{\circ }}{\mathrm{mm}}\right)$	${(}^{\circ })$	${(}^{\circ })$	(m${}^{-1}$)		
Direction 1	11	−41.0	46.8	376	−2.34	0.81
Direction 2	11	−91.2	47.4	376	−0.65	0.80
Direction 3	11	−108.7	47.2	374	−0.23	0.81
